# Decoding repetitive finger movements with brain activity acquired via non-invasive electroencephalography

**DOI:** 10.3389/fneng.2014.00003

**Published:** 2014-03-13

**Authors:** Andrew Y. Paek, Harshavardhan A. Agashe, José L. Contreras-Vidal

**Affiliations:** Laboratory for Non-invasive Brain-Machine Interface Systems, Department of Electrical and Computer Engineering, University of HoustonHouston, TX, USA

**Keywords:** electroencephalography, EEG, hand, finger, decoding

## Abstract

We investigated how well repetitive finger tapping movements can be decoded from scalp electroencephalography (EEG) signals. A linear decoder with memory was used to infer continuous index finger angular velocities from the low-pass filtered fluctuations of the amplitude of a plurality of EEG signals distributed across the scalp. To evaluate the accuracy of the decoder, the Pearson's correlation coefficient (*r*) between the observed and predicted trajectories was calculated in a 10-fold cross-validation scheme. We also assessed attempts to decode finger kinematics from EEG data that was cleaned with independent component analysis (ICA), EEG data from peripheral sensors, and EEG data from rest periods. A genetic algorithm (GA) was used to select combinations of EEG channels that maximized decoding accuracies. Our results (lower quartile *r* = 0.18, median *r* = 0.36, upper quartile *r* = 0.50) show that delta-band EEG signals contain useful information that can be used to infer finger kinematics. Further, the highest decoding accuracies were characterized by highly correlated delta band EEG activity mostly localized to the contralateral central areas of the scalp. Spectral analysis of EEG also showed bilateral alpha band (8–13 Hz) event related desynchronizations (ERDs) and contralateral beta band (20–30 Hz) event related synchronizations (ERSs) localized over central scalp areas. Overall, this study demonstrates the feasibility of decoding finger kinematics from scalp EEG signals.

## Introduction

Understanding how the human brain controls hand movements presents an interest to researchers in neuroscience, engineering, and robotics because of the hand's usefulness and its inherent complexity in its multiple degrees of freedom that provides its multi-functionality (Schieber and Santello, 2004; Ingram et al., 2008). Numerous neural activity recording techniques have been employed with humans and primates in order to elucidate the neural mechanisms behind hand control.

Functional magnetic resonance imaging studies (fMRI) have suggested that the movement of each finger is represented in separate somatotopic, but largely overlapping, areas of the supplementary motor area (SMA) and the primary motor cortex (M1) of the human brain (Beisteiner et al., 2001; Indovina and Sanes, 2001), in such a manner that the SMA activates before the M1 during finger movements (Wildgruber et al., 1997). fMRI has also been used to demonstrate that ipsilateral cortical regions are involved in sequential imaginary hand movements (Ueno et al., 2010). It has also been suggested with fMRI that cerebellar regions are activated during finger movements that are paced in time from memory as opposed to visual cues (Kawashima et al., 2000). fMRI has also been coupled with scalp electroencephalography (EEG) to estimate current sources associated with finger movements, which were found not only in the central sulcus, but also in frontal medial and parietal regions (Ball et al., 1999).

Scalp EEG studies have also investigated how neural rhythms are associated with hand movements. Event-related desynchronizations (ERDs) and event-related synchronizations (ERSs) have been characterized respectively as decreases and increases in power at various frequency bands. ERDs have been found in alpha (8–12 Hz) rhythms during the execution of hand movements while ERSs have been found in beta (12–24 Hz) rhythms when hand movements stop. Such alpha ERDs and beta ERSs were typically found in central areas of the scalp contralateral to hand movements (Pfurtscheller et al., 1998; Pfurtscheller and Lopes da Silva, 1999). These alpha ERDs and beta ERSs were also found to be more pronounced in faster hand clenching movements (Yuan et al., 2010). Readiness Potentials (RPs) have also been characterized with hand movements in EEG studies as a slow increase in negativity that occurs before movement onset (Babiloni et al., 1999; Cui and MacKinnon, 2009). Such RPs have been shown with EEG to change in timing due to planning of different types of sequential finger movements (Bortoletto et al., 2011) and become more pronounced in self-initiated movements (Gerloff et al., 1998a; Cui and MacKinnon, 2009). Gerloff et al. (1997) also characterized from EEG steady-state movement related cortical potentials (SSMRCPs) with repetitive finger movement.

Magnetoencephalography (MEG) has been used with current source localization techniques to identify areas associated with finger movements. Gerloff et al. (1998b) characterized peaks in magnetic field and current sources occurring on and after movement onsets. The estimated sources were found to evolve from the anterior area of the contralateral central sulcus toward the posterior area of the central sulcus during movement (Gerloff et al., 1998b). Current sources have also been found in the ipsilateral premotor areas, which peak in amplitude earlier than sources found in the contralateral motor areas (Huang et al., 2004). Current sources have also been studied with active and passive finger movements (Onishi et al., 2013) and finger movements paced with acoustic stimuli (Pollok et al., 2004; Boonstra et al., 2006).

Finger movements have also been studied with neural activity recorded with microelectrodes implanted in monkey cortical tissues in the motor area. It was found that single neurons modulate their firing rate depending on what combination of fingers moved and whether they flexed or extended (Schieber and Hibbard, 1993). Poliakov and Schieber (1999) have also shown that using clustering approaches with single unit activity from neurons only yield limited groupings, suggesting that neurons act in a very diverse manner and act as a network in controlling finger movements. In this regard, neural decoding approaches have been used with neural spiking activity to demonstrate that the activity from a population of neurons can be used as a linear combination to predict finger movements. Such studies have found high accuracies in classifying which finger was moved and whether the finger extended or flexed (Hamed et al., 2007; Acharya et al., 2008). Grasp postures and wrist orientations have also been decoded from neural discharge rates (Townsend et al., 2011). Firing rates and local field potentials (LFPs) recorded with microelectrodes have also been used to decode the time course of arm and finger movements during grasp movements (Vargas-Irwin et al., 2010; Zhuang et al., 2010; Bansal et al., 2011; Liang and Bougrain, 2012) and individual finger movements (Aggarwal et al., 2009).

Hand movements have also been decoded with brain rhythms recorded on a larger spatial scale with electrocorticography (ECoG) in human epilepsy patients. Neural decoding approaches show that the time course of the individual fingers can be reconstructed when subjects were called to flex individual fingers (Kubánek et al., 2009; Liang and Bougrain, 2012), and perform slow deliberate grasping motions (Acharya et al., 2010).

Relatively few studies have shown that non-invasive neuroimaging techniques can be used to predict hand movements. Gallivan et al. (2011) demonstrated classifying different grasp gestures with fMRI. Quandt et al. (2012) have shown that MEG and scalp EEG can be used to classify which finger was moved and whether they flexed or extended. Our previous work demonstrated the use of EEG in predicting trajectories of angular finger joint movements during grasping (Agashe and Contreras-Vidal, 2011). Table 1 summarizes hand grasping and finger decoding studies.

**Table 1:** **Summary of finger movement decoding studies**.

**Behavioral task**	**Decoded kinematics**	**Decoding accuracy (Pearson's correlation coefficient)**	**Signal modality; features; subjects**	**References**
3D Reach-to-grasp	Finger joint angles	Monkey C: *r* = 0.72 Monkey G: *r* = 0.74 (medians)	Microelectrode; neuron firing rates; Monkeys C, G	Vargas-Irwin et al., 2010
3D Reach-to-grasp	Grasp aperture	Delta: *r* = 0.46 Gamma: *r* = 0.62 (average)^*^	Microelectrode; LFP data; Monkeys C, G	Zhuang et al., 2010
3D Reach-to-grasp	Grasp aperture	Position: *r* = 0.65 Velocity: *r* = 0.75 (averages)	Microelectrode; 0.3–2 Hz LFP data; Monkeys C, G	Bansal et al., 2011
Individual finger flexion and extension	Strain gauge measurement time traces	Thumb: *r* = 0.88 Index: *r* = 0.81 Middle: *r* = 0.82 Ring: *r* = 0.84 Little: *r* = 0.86 (averages)	Microelectrode; neuron firing rates; Monkeys C, K	Aggarwal et al., 2009
Slow and deliberate grasping task	Principle component of finger joint angles	*r* = 0.51 (median)	ECoG; 2 s moving average filter; 4 human patients	Acharya et al., 2010
Repetitive individual finger flexion and extension	Individual finger flexion traces	Thumb: *r* = 0.56 Index: *r* = 0.60 Middle: *r* = 0.54 Ring: *r* = 0.50 Little: *r* = 0.42 (averages)	ECoG; 100 ms average window; frequency bins from 8 to 175 Hz; 5 human patients	Kubánek et al., 2009
Individual finger flexion	Individual finger flexion traces	Thumb: *r* = 0.59 Index: *r* = 0.51 Middle: *r* = 0.32 Ring: *r* = 0.53 Little: *r* = 0.42 (averages)	ECoG; 3 frequency bins from 1 to 200 Hz; 3 human subjects	Liang and Bougrain, 2012
3D Reach-to-grasp	MCP joint angles	*r* = 0.76 (averaged across all fingers)	EEG; 0.1–1 Hz delta rhythms; 5 human subjects	Agashe and Contreras-Vidal, 2011
Repetitive finger taps	Index finger MCP joint angle	*r* = 0.36 (median)	EEG; 0.1–3 Hz delta rhythms; 5 human subjects	Current study

The table indicates which behavioral task was performed, what kinematics were reconstructed from neural activity, the decoding accuracies that measured how well the reconstructed finger movements matched the observed movements, the signal modality and subjects used in the study.

* Accuracies correspond to the use of delta (0.3–4 Hz) and high gamma (200–400 Hz) frequency bands to decode grasp aperture.

While there are numerous neuroimaging studies which have studied finger movements, they typically involved characterizing grand averages with respect to the onset of movement. However, it is also of interest to study how neural activity may be related to the timing and amplitude of finger movements throughout the movement execution. Neural decoding approaches that reconstruct the time course of finger movements can help explore such relationships through data driven models. While neural decoding approaches with single unit activity has helped elucidate how neurons may infer movement as a population, decoding on the macroscale could help elucidate how broad networks across the brain could be related to finger movements. Indeed, macroscale neuroimaging studies suggest that not only the primary motor area is involved with finger movements, but ipsilateral, frontal, and parietal areas are also activated. As an advantage over grand average approaches, experiments involving decoding approaches could allow for more varied and natural behavioral tasks because decoding only relies upon the synchronization between the neural activity and the kinematics of interest. Establishing the relationship between neural signals and finger movements could also be used to drive commands in hand based neuroprosthetic devices or brain machine interfaces (Hochberg et al., 2006). Thus, in this study we investigate whether it is possible to infer finger kinematics from scalp EEG.

We hypothesize that finger movements can be decoded from scalp EEG signals based on the rationale that delta band EEG signals could be used to reconstruct finger movements. Previous studies indicate that detailed information about finger movement is carried in amplitude modulations of the smoothed ECoG or LFP signals in the delta (0.1–4 Hz) band (Kubánek et al., 2009; Acharya et al., 2010; Zhuang et al., 2010; Bansal et al., 2011; Liang and Bougrain, 2012). Although EEG recordings represent the activity from large and separated groups of neurons (Krusienski et al., 2011), it can be argued that these amplitude modulations can also be recorded from EEG as low-frequency delta band signals. Previous studies with MEG and EEG have also shown time locked peaks in electrical potentials and magnetic fields associated with the onset of finger movements which also evolve slowly (Gerloff et al., 1997, 1998b). It is also favorable to use EEG signals in the delta band because they have more power (i.e., the EEG power spectrum follows a 1/f pattern) and are also less likely to be affected by frequency-dependent signal propagation through the brain, skull, cerebral spinal fluid, and the skin than higher frequency components. Delta band EEG signals are also less likely to be affected by muscular artifacts (Goncharova et al., 2003; Fatourechi et al., 2007). In this “proof-of-principle” study, a linear decoder with memory was embedded within a genetic algorithm (GA) to infer the angular velocity of the metacarpal-phalangeal (MCP) joint of the index finger from the derivative of the EEG signals.

## Methods

### Recording and behavioral task

Five able-bodied right-handed subjects participated in this study (age 25 ± 2 years, 4 males and 1 female) and gave informed consent as approved by the University of Maryland Institutional Review Board. Subjects were instructed to tap their right index finger three times in succession while seated behind a table with their forearms comfortably resting flat on the table. Each trial (consisting of a series of three taps) was self-initiated. EEG and hand kinematics were recorded simultaneously while subjects performed the finger tapping task. EEG signals were recorded over the entire scalp using a 64 channel HydroCel Geodesic Sensor Net (Electrical Geodesics, Inc., Eugene, Oregon). The recorded EEG signals were amplified and digitized at 500 Hz with Net Amps 300 (Electrical Geodesics, Inc., Eugene, Oregon). Trajectories of 18 joint angles were recorded with a wireless data glove (CyberGlove, CyberGlove Systems LLC, San Jose, California) at a resolution of 0.93° at a non-uniform sampling rate of 35–70 Hz. The glove was calibrated once for each subject by manually adjusting the gain and offset of each glove sensor's raw value and by visually verifying that the joint angles between the virtual hand and the actual hand matched. Subjects were recorded for ~20 min each, which recorded ~100–200 trials. The first 100 trials that were completed correctly (where subjects did not tap more or less than three times) were used in the following decoding steps.

To synchronize the EEG recordings with the kinematics recordings, a video of the session was recorded at 30 frames per second. The video was synchronized with the EEG internally by the recording software (NetStation 4.3, Electrical Geodesics Inc.). Both the video and the data glove software simultaneously recorded the on and off status of a red LED, which was mounted on the glove. Manually turning the LED on and off three times consecutively served as event markers to synchronize the glove data and the video, and thus the EEG as well. The raw synchronized EEG and kinematics recordings were then resampled at 100 Hz in the following manner. A Chebychev II antialiasing filter at 40 Hz was applied to the raw EEG signals followed by a down-sampling to 100 Hz. The raw kinematics signals were interpolated with a piecewise cubic hermite interpolating polynomial and up-sampled to 100 Hz.

### Kinematics analysis

To observe the variation of the finger tapping motion across subjects, different measures of the finger tapping motion were calculated for each trial including: trial length, tapping speed, resting position, extension angle, and range of motion (ROM). The average, standard deviation, and coefficient of variance of these measures were calculated across all trials for each subject. The statistics of these measures are shown in Table 3.

The temporal statistics of the finger tapping motion were measured by calculating the trial length and tapping speed of each trial. The trial duration was approximated by locating when the tapping motion started, called the movement onset time, and locating when the tapping motion ended, called the movement offset time. Movement onsets were determined to be time points when the joint angle speed exceeded 5% of the maximum velocity for the first time during a trial. Similarly, movement offsets were determined to be time points when the joint angle speed was within 5% of the maximum speed for the last time near the end of the trial. Subtracting the movement onset time from the movement offset time of each trial yielded the trial length. For each trial, dividing the number of taps (three) by the trial length yielded the approximate tapping speed.

The angular statistics of the finger tapping motion were measured by calculating the resting position, the extension angle, and the ROM of each trial. The resting position of each trial was estimated by calculating the average of the finger positions in 1 s segments before movement onset and after movement offset. The extension angle of each trial was taken as the average of the three local maxima in the finger trajectories. The ROM of each trial was calculated by subtracting the resting position from the extension angle.

The power spectral density (PSD) of the kinematics was calculated for the continuous finger trajectory data. First, the index finger trajectory data across the recording session containing the first 100 trials was detrended. The PSD of the data was calculated by using the Thomson Multitaper method PSD function in MATLAB (MathWorks, Inc., Natick, Massachusetts). A time bandwidth product of 4 and the Fourier transform window length of 512 were used. The frequency below which captured 95% of the cumulative power in the PSD is shown in Table 3.

### Decoding kinematics from EEG

The following describes how the EEG signals were used to decode finger movements.

#### Preprocessing

**Figure 1:**
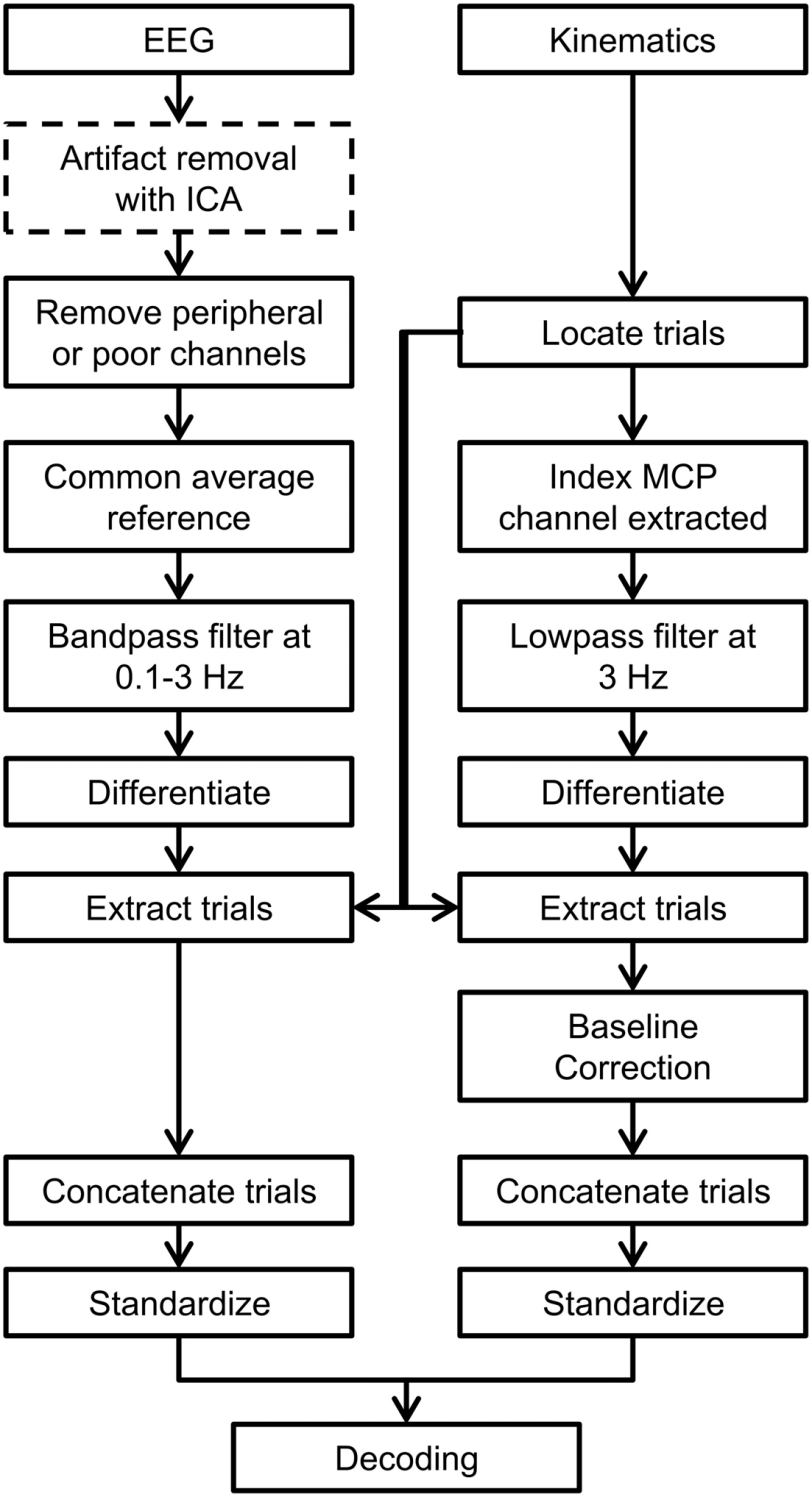
**Flow chart indicating the preprocessing steps used in this study**. ICA-based artifact removal is shown as a dotted line in the preprocessing steps as it was only used in one decoding scenario when artifacts were removed from EEG.

Before designing and calibrating the decoder using the index finger's trajectories and the EEG data, both data sets were pre-processed. A flow chart of the pre-processing steps is shown in Figure 1.

**Figure 2:**
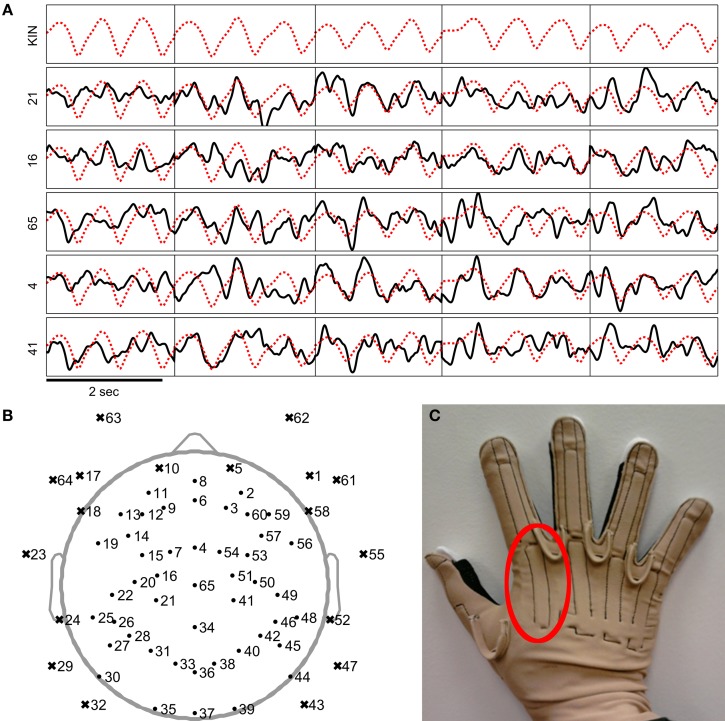
**Examples of the preprocessed EEG and kinematics signals used in the study. (A)** The EEG signals are plotted as a black line while the index finger MCP joint velocity trajectory is plotted as a dotted red line. Signals were preprocessed as mentioned in the steps above. (Since preprocessed EEG was negatively correlated to the kinematics, the plotted traces were negated to help illustrate similarities between the EEG and kinematic traces). The vertical black lines indicate where the segmented trials begin and end. **(B)** Locations of the EEG channels are plotted as shown. Channels marked by a dot and an x-shaped mark were respectively included and removed from the study. **(C)** Photograph of the data glove apparatus (CyberGlove, CyberGlove Systems LLC, San Jose, California) used in the study. The area in the red circle indicates the sensor used to record the angles of the index MCP joint.

First, EEG signals from 18 peripheral channels along frontal and temporal sites were rejected. The rejected channels are shown as x-shaped markers in Figure 2B. These channels were removed because they were most likely to be strongly influenced by artifacts such as sensor movements due to facial gestures, eye movements, or muscular activity. Further EEG data from the remaining channels were also visually inspected and rejected if they contained large and isolated changes in amplitude which were likely caused by poor impedance or isolated sensor movements. These sensors that were manually removed are indicated in Figure 7. Then, the EEG signals were common average referenced and the EEG recordings were then high pass filtered at 0.1 Hz with a zero-phase 8th order Butterworth filter. Only the (task-relevant) data from the MCP joint of the index finger was used in the study, so the kinematics data from the other channels were removed. The sensor associated with the MCP joint of the index finger is indicated in Figure 2C.

Next, both EEG and kinematics recordings were low-pass filtered at 3 Hz (i.e., within the delta band) with a zero-phase 1st order Butterworth filter. The EEG signals were low pass filtered to extract the relevant delta band signals that we predict to be correlated to movements. The kinematic signals were low-pass filtered to smooth the step-like structure introduced by the glove's resolution limits. The low pass cut off frequency of 3 Hz was chosen as this frequency was found to retain more than 95% of the cumulative power in the finger movement PSD across all subjects. Low pass filtering the finger movements at 3 Hz was also found to reasonably preserve the integrity of the tapping trajectories upon visual inspection.

After filtering, the EEG and kinematics were transformed into their derivatives. Using the derivative of the EEG and kinematics in preliminary decoding attempts was found to increase decoding accuracy. This improvement may have occurred because the use of derivatives inherently processes the signals (where the mean or very low frequency drifts throughout the signals were suppressed) and/or may indicate increased relevance of positional changes in a more dynamic neural representation of limb movements (Bradberry et al., 2009; Bansal et al., 2011).

The continuous EEG and kinematics were then extracted into segments consisting of the movement period from 0.1 s before movement onset to 0.1 s after movement offset (as described in section Kinematics Analysis). Any data outside of the movement periods were not used for further analysis. The segmentation allowed the decoder to be calibrated using the kinematics that only contained variations due to movement. The segmented kinematics data were baseline corrected by the mean of the segment −0.1 to 0 s with respect to movement onset. This was done to reduce the effects of the very slow changes in magnitudes found in the kinematics data recorded by the data glove throughout the recording session. After segmenting, the data was concatenated and standardized with respect to the means of each channel. Examples of the preprocessed data are shown in Figure 2A.

#### Linear decoder

A linear decoder was used to predict the index MCP joint angular velocity from the derivative of the EEG signals. The overall paradigm involved using the magnitudes from EEG signals from certain channels at different temporal points in the past to calculate the present joint angular velocity. EEG sensors to be chosen for decoding were selected through a GA that found an optimal set of channels that maximized decoding accuracies (see section Channel Selection through the GA for further details). The index MCP joint angular velocities were modeled as a linear combination of data from the selected sensors:

$$\theta^{\prime }(t)=\sum_{i=1}^{N}\sum_{k=1}^{L}b_{ik}{S^{\prime }}_{i}\;(t\;-\;\tau_{k})$$

where θ'(*t*) is the angular velocity of the index MCP joint at time *t*, *i* corresponds to a certain *i*-th sensor where *N* is the total number of sensors (*N* = 47), *k* corresponds to the *k*-*th* temporal lag, τ_*k*_, which creates an offset between the EEG and kinematics signal where *L* is the total number of lags or embedding dimension (*L* = 7), *b*_*ik*_ is the weight which is the coefficient that is multiplied by the magnitude of the *i*-th sensor at a certain time lag *k*, *S*^'^_*i*_(*t* − τ_*k*_) is the magnitude of the EEG sensor's derivative from *i*-th sensor at time *t* − τ_*k*_, and *t* is the time in seconds. The data was decoded with multiple lags where τ_*k*_ = 0, 50, 100, 150, 200, 250, and 300 ms in the past.

#### Model training and validation

**Figure 3:**
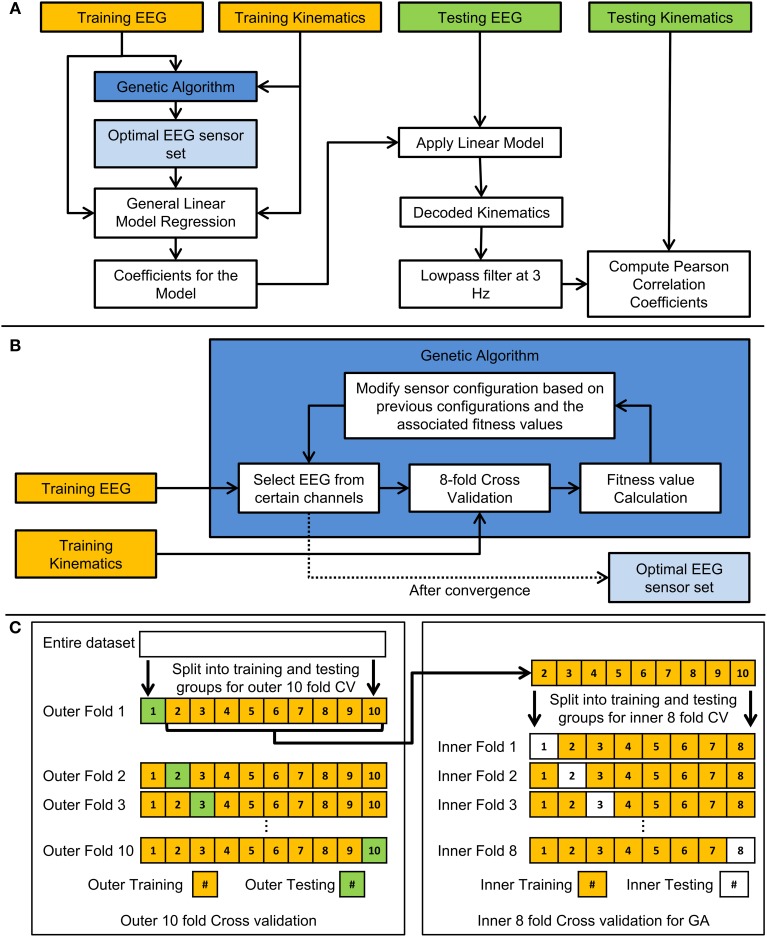
**Flow chart of the cross-validation and genetic algorithm schemes. (A)** Flow chart demonstrating how the 10-fold cross validation was performed to train and test the linear model. **(B)** Flow chart indicating how the genetic algorithm was employed in this study. **(C)** Diagram indicating how the entire data set is arranged for the two types of cross validation used in the study. The outer 10-fold cross validation was used to test the generalizability of the linear model and optimal EEG sensor set. The inner 8-fold cross validation was used to validate the performance of various EEG sensor combinations in a genetic algorithm (GA) optimization.

The performance of the decoder was evaluated using the 10-fold (outer) cross validation scheme. Each fold presented a situation where the decoder was calibrated with a set of EEG and finger movement data and then tested to observe how well the decoder performed with novel EEG signals. The flow chart for the scheme is shown in Figure 3A. 100 trials were used to train and test the linear decoder for each subject. For each fold, the data was split into 10 groups, 9 of which were used to train the linear decoder while the remaining group was used to test the decoder. Each group consisted of 10 trials. The training set was used with the GA to find the optimal set of EEG channels to use with the linear model. After the optimal set of EEG sensors were found, the training EEG data from such sensors were used. The weights *b*_*ik*_ of each channel from each lag were calculated as the coefficients which fit a generalized linear model (GLM) between the EEG signals and the kinematics signals from the training trials. The weights *b*_*ik*_ were calculated using GLM functions in MATLAB (MathWorks, Inc., Natick, Massachusetts). Using the calculated weights from the training data, the linear decoder was used with the EEG data from the testing group to predict the observed joint velocities in the same testing group. The predicted trajectories were then standardized and low-pass filtered. The decoding accuracy was calculated as the Pearson correlation coefficient (*r*-value) between the concatenated predicted trajectories and the concatenated observed trajectories in the testing set. This was repeated for each fold, each of which consisted of different training and testing groups of trials (i.e., cross-validation).

#### Channel selection through the genetic algorithm (GA)

Previous decoding studies have found that an optimal number of electrodes or neurons were needed to increase the decoding accuracy of the trajectories of hand movements (Hamed et al., 2007; Bradberry et al., 2009; Acharya et al., 2010). In a previous study from our lab, we found that decoding accuracies begin to decrease if more than an optimal set of channels were used, possibly due to over-fitting of data in the training sets (Bradberry et al., 2009). In this study, we decided to explore the use of the GA to find an optimal set of channels to be used in decoding. GAs have been used in EEG studies to improve the detection of neural pathologies such as Alzheimer's Disease and Epilepsy (Kim et al., 2005; Ocak, 2008). GAs have also been used to improve classifiers that detect motor intent for BCI application by improving the quality of trials used to train the classifier (Wang et al., 2012), or by determining which channels are used with the classifier (Wei et al., 2010). In the context of this study, the GA was utilized to find the optimal set of channels that yielded the highest decoding accuracies. Further details on GAs can be found in (Mitchell, 1998; Haupt and Haupt, 2004).

**Figure 4:**
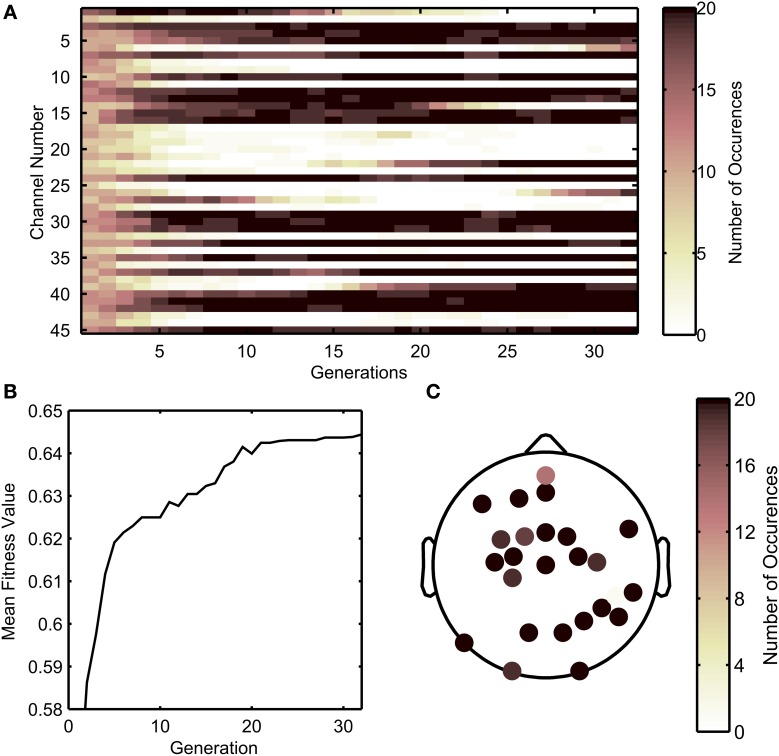
**Example of the progression of the genetic algorithm, showing the evolution of channels selected across generations. (A)** Number of times each channel was selected across individuals from each generation. Darker colors indicate that more individuals within a generation selected that particular channel. **(B)** The median fitness value across all individuals in a population increases through the progression of generations. **(C)** Scalp map depicting how many times a sensor is selected by all individuals in the last generation for the sample run. As shown in **(A)**, darker channels indicate that more individuals in the last generation selected a channel.

Figure 3B shows a flow chart of how the GA was implemented. The decoding process involved an inner cross-validation step and an outer cross validation (testing for generalization) step, in which the optimal channel set uncovered by the GA algorithm acting on a training data set (comprised of EEG and kinematics), was tested on unseen testing data in the outer cross validation loop. The GA in this study was designed to find the optimal combinations of EEG channels to include in the decoding. To briefly describe the process, the GA framed the genes of individuals in a population as a set of EEG sensors. First, 8-fold (inner) cross validation was performed with each individual, and its fitness value was calculated as the median of *r*-values across the 8-folds. New individuals in the next generation were derived through cloning, cross over, or mutations with individuals from the previous generation that had relatively high fitness values. This process was repeated until the best fitness value did not improve substantially across generations (where the weighted average of the best fitness value across the previous 30 generations did not improve by 0.01). Technical details regarding the GA can be found in the MATLAB references (MathWorks, Inc., Natick, Massachusetts). Relevant technical parameters used in the GA are shown in Table 2. An example of the how the population evolved throughout the generations is shown in Figure 4. Initially, each individual contained a random set of EEG sensors. As the GA progressed through the generations, the decoding accuracies of the populations increased, and most of the individuals in the population converged to a particular set of EEG sensors.

**Table 2:** **Parameters used in the Genetic Algorithm**.

**Parameter**	**Set value**
Population type	Bit string
Population size	20
Creation function	Uniform
Crossover function	Crossover scattered
Crossover fraction	0.5
Elite count	2
Mutation function	Uniform
Mutation rate	0.01
Fitness value	Median correlation coefficient across 8-folds
Fitness scaling function	Rank
Selection function	Stochastic
Stall generations	30
Function tolerance	0.01
Maximum number of generations	100

Note that the inner 8-fold cross validation used in the GA served a different purpose from the outer 10-fold cross validation. This inner 8-fold cross validation was repeated numerous times in one GA optimization in order to validate the performance of decoding with various combinations of EEG channels. One GA optimization took place in each fold in the outer 10-fold cross validation. The outer 10-fold cross validation was used to test the generalizability of the optimal sensor set and the linear model with testing data that was unseen in the GA or in the linear regression. Figure 3C shows an example of how the data was arranged for the inner 8-fold cross validation from a single fold in the outer 10-fold cross validation.

#### Assessing the effects of artifactual components

The decoding process was repeated three more times with modified EEG data to ascertain if the decoding did not result from spurious sources. In the first scenario, artifacts were removed from EEG with independent component analysis (ICA). ICA has been used previously to remove artifacts from EEG (Vigário, 1997; Fatourechi et al., 2007). Components which resembled ocular artifacts, head movements, or brief sensor movements were removed from EEG. (Components which may have resembled EMG activity were not observed). ICA was performed through the EEGLAB toolbox (Delorme and Makeig, 2004). In the second scenario, EEG that was only collected from the peripheral sensors was used for decoding. (These sensors were removed previously due to their susceptibility to artifacts). Common average referencing was not used as this would have caused artifacts such as eye movements to spread to data from other peripheral sensors. In the third scenario, EEG data from the rest period before the movements was used to decode finger movements. For brevity, we refer to the four decoding scenarios as decoding with unmodified EEG, ICA-pruned EEG, peripheral EEG, and rest EEG.

### Ancillary EEG analyses

#### Alpha and beta band power change analysis

In order to help ascertain which spatial areas of the scalp may be involved in finger movements, the spatial distribution of alpha ERDs and beta ERDs were calculated. EEG data from peripheral and poor channels were removed as indicated in section Preprocessing. The EEG was common average referenced and then segmented with respect to movement onsets and offsets in the finger kinematics. Each trial was segmented into three segments: rest, movement, and end. The rest period contained data −1.5 to −0.5 s with respect to the movement onset. The movement period contained data 0.5–1.5 s after movement onset. The end period contained data 0–1 s after movement offset. The time periods are shown in Figure 8. Then, the EEG signals within each of these time periods were detrended. For each of these periods, the PSD of the EEG signal was calculated using the Thomson Multitaper method in MATLAB with a Fourier transform length of 512 and a time bandwidth product of 4. The Thomson Multitaper method was chosen as it helps reduce the variance and spectral leakage associated with estimating the PSD with periodograms in a non-parametric manner (Thomson, 1982). Previous work in EEG classification studies have used the Thomson Multitaper method to extract spectral features based on these advantages (Mensh et al., 2004; Herman et al., 2008; Alipoor et al., 2010). The alpha ERD for the trial was calculated as the relative change in power in the alpha band (8–13 Hz) from the rest to movement periods. The beta ERS for the trial was calculated as the change in power in the beta band (20–30 Hz) from the movement to end periods. This process was done for each trial, yielding 100 alpha ERD and beta ERS values for each channel. The sign test was used to determine if the distribution of alpha ERDs and beta ERSs across trials for each channel were significantly different from a distribution with a median of zero. The sign test was used as the alpha ERD and beta ERD distributions tended to have a skewed distribution across trials.

#### Spatial distribution of correlated delta band EEG

In order to ascertain which spatial areas of the scalp may have contributed to the decoding of finger movements, the correlation coefficients between the preprocessed EEG data from each channel and each time lag and the preprocessed kinematics data was calculated for each trial. This was done for each trial, yielding 100 correlation coefficient values for each channel and for each lag. The sign test was used to ascertain if the distribution of the correlation coefficients across all trials for each EEG sensor and lag were significantly different from a distribution with a median of zero.

#### Delta band and feature ensemble averaging

To study the temporal characteristics of the delta band EEG that were used to decode finger movements, grand averages of the preprocessed delta band EEG and finger trajectory data were calculated. The EEG and finger trajectory data were preprocessed as indicated in section Preprocessing with some exceptions. EEG and kinematics were segmented −1.5 to 3 s with respect to the movement onset of the finger movements. The EEG and kinematics signals were also baseline corrected by subtracting the mean of the signal −1.5 to −0.5 s with respect to movement onset. In one case, the derivative was not used to compare grand averages between delta band EEG with finger position. In another case, the derivative was used to compare the grand averages between derivative delta band EEG with finger velocities. The grand averages of the EEG and kinematics were then standardized with respect to their means.

## Results

### Kinematics statistics

Table 3 shows the statistics of the tapping task performed by the subjects. We note the wide variability across subjects in the full ROM and in the tapping speeds. The calculated tapping speeds as well as the PSD suggest most of the variation in the raw finger trajectories was contained below 3 Hz, justifying the low pass filter cut off frequency for both the EEG and the finger kinematics in the preprocessing steps.

**Table 3:** **Statistics of the index MCP finger movements**.

**Subject**	**Extension (degrees)**	**Rest (degrees)**	**ROM (degrees)**	**Trial length (sec)**	**Tapping speed (taps/sec)**	**Frequency of 95% cumulative PSD (Hz)**
1	17.31	−22.33	39.64	1.43	2.11	2.54
	(4.21)	(5.54)	(4.46)	(0.12)	(0.17)	
	0.24	0.25	0.11	0.08	0.08	
2	9.45	−25.20	34.65	1.27	2.37	2.34
	(6.03)	(6.35)	(4.16)	(0.10)	(0.17)	
	0.64	0.25	0.12	0.08	0.07	
3	14.56	−6.96	21.52	2.12	1.42	1.56
	(2.74)	(2.77)	(3.26)	(0.15)	(0.10)	
	0.19	0.40	0.15	0.07	0.07	
4	24.63	−22.39	47.01	1.93	1.59	1.76
	(3.69)	(1.00)	(3.53)	(0.30)	(0.23)	
	0.15	0.04	0.08	0.16	0.14	
5	27.82	−17.08	44.90	2.44	1.25	1.56
	(2.71)	(2.28)	(2.80)	(0.28)	(0.13)	
	0.10	0.13	0.06	0.11	0.10	
All	18.76	−18.79	37.55	1.84	1.75	1.95
	(7.81)	(7.67)	(9.82)	(0.48)	(0.46)	(averaged)
	0.42	0.41	0.26	0.26	0.26	

The data glove apparatus measures the index MCP joint angle such that when the index finger is parallel with the palm, the joint is measured as 0 degrees. When the finger extends away from the palm, it is measured in positive degrees. When the finger flexes toward the palm, it is measured in negative degrees. For each value, the mean is given at the top, the standard deviation is shown in the middle in parenthesis and, and the coefficient of variance is shown at the bottom with an underlined value.

### Decoding accuracies varied across subjects

**Figure 5:**
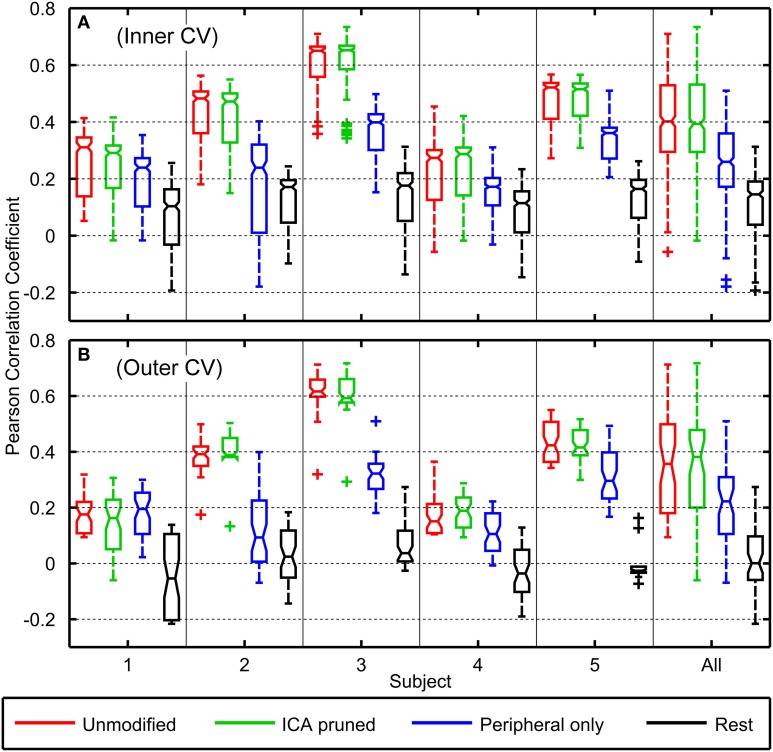
**Boxplots of Pearson correlation coefficients of the decoder's predicted trajectories against the observed trajectories for all subjects**. Boxplot **(A)** represents accuracies across the inner cross validation folds (*n* = 80) while boxplot **(B)** represents accuracies across the outer cross validation folds (*n* = 10). The boxplots on the right describes the distribution of *r*-values from all subjects. Red, green, blue, and black boxplots respectively correspond to decoding accuracies where unmodified EEG, EEG with ICA-based artifact removal, EEG from only peripheral sensors, and EEG from the rest period were used in the neural decoding.

Figure 5 depicts the decoding accuracies from the *inner* 8-fold (Panel A) and *outer* 10-fold (Panel B) cross validation schemes. Considering the outer cross validation, the decoding performance using unmodified EEG varied across subjects with a median of *r* = 0.36 and a maximum of *r* = 0.71 (minimum *r* = 0.09, lower quartile *r* = 0.18, upper quartile *r* = 0.50). The highest median decoding accuracy was obtained for subject 3 (median *r* = 0.62) who had the smallest range of finger motion (ROM = 21.52°) and the lowest accuracy was observed for subject 4 (median *r* = 0.15) who had the largest finger excursions (ROM = 47.01°).

Using ICA-pruned EEG yielded slightly higher decoding accuracies with a median *r* = 0.38 and a maximum of *r* = 0.72 (minimum *r* = −0.06, lower quartile *r* = 0.20, upper quartile *r* = 0.48). Using peripheral EEG channels yielded substantially poorer results, where the performance across subjects had a median of *r* = 0.22, and a maximum of *r* = 0.51 (minimum *r* = −0.07, lower quartile *r* = 0.11, upper quartile *r* = 0.31). Using rest EEG to decode movements yielded the poorest accuracies across subjects with a median of *r* = 0.00, and a maximum of *r* = 0.27 (minimum *r* = −0.22, lower quartile *r* = −0.06, upper quartile *r* = 0.10). Based on the Kruskal–Wallis Test, the *r*-values from unmodified EEG decodings were not statistically significantly different from ICA-pruned EEG decodings (*p* = 0.98). Pairwise comparisons showed *r*-values from peripheral EEG decodings and rest EEG decodings were statistically significantly different from all other conditions (*p* < 0.001).

Decoding accuracies from the inner cross validations were generally found to be slightly higher than those of the outer cross validations. Across subjects, decoding with unmodified EEG data yielded a median *r* = 0.40, ICA-pruned EEG data yielded a median of *r* = 0.39, peripheral EEG data yielded a median *r* = 0.26, and rest EEG data yielded a median of *r* = 0.15.

**Figure 6:**
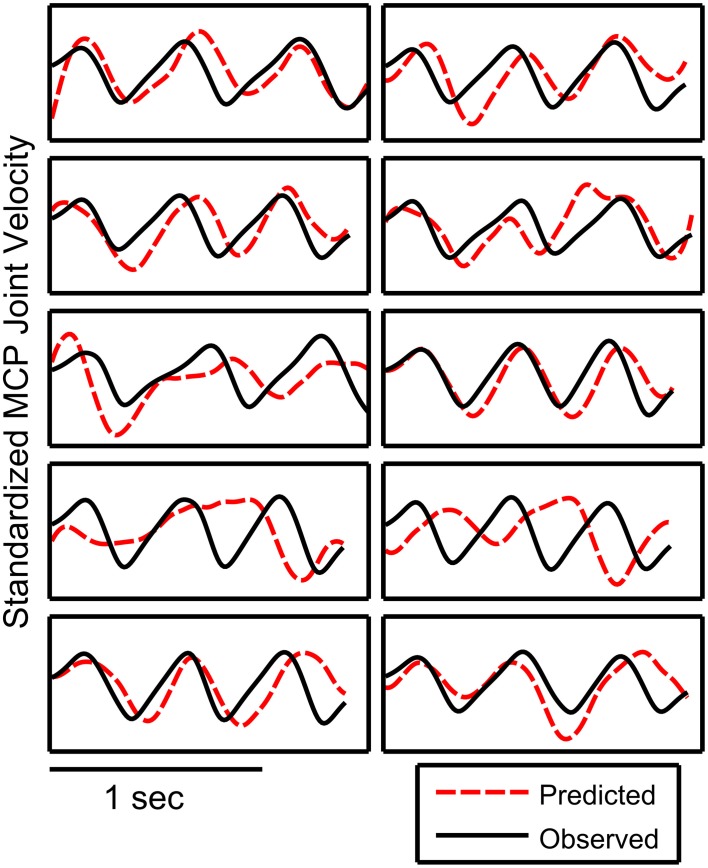
**Examples of observed and predicted trajectories**. This example was taken from the fold with the decoding performance (*r* = 0.37, subject 2) that resembled the median decoding acuracies across all subjects. The decoding accuracy for a fold was calculated as the correlation coefficient between the observed and predicted trajectories when the 10 trials are concatenated. The decoded trajectory is shown as a dotted red line while the observed trajectory is plotted as a solid black line. The plotted trajectories were standardized and low pass filtered.

Figure 6 shows examples of the observed and predicted joint velocity kinematics for a single fold where the accuracies are close to the median across all subjects. This example from subject 2 shows how the predicted finger movements mostly follow the observed trajectories, however there were a few trials and time periods where the predicted movements do not match.

### Optimal channel set selections varied across subjects

**Figure 7:**
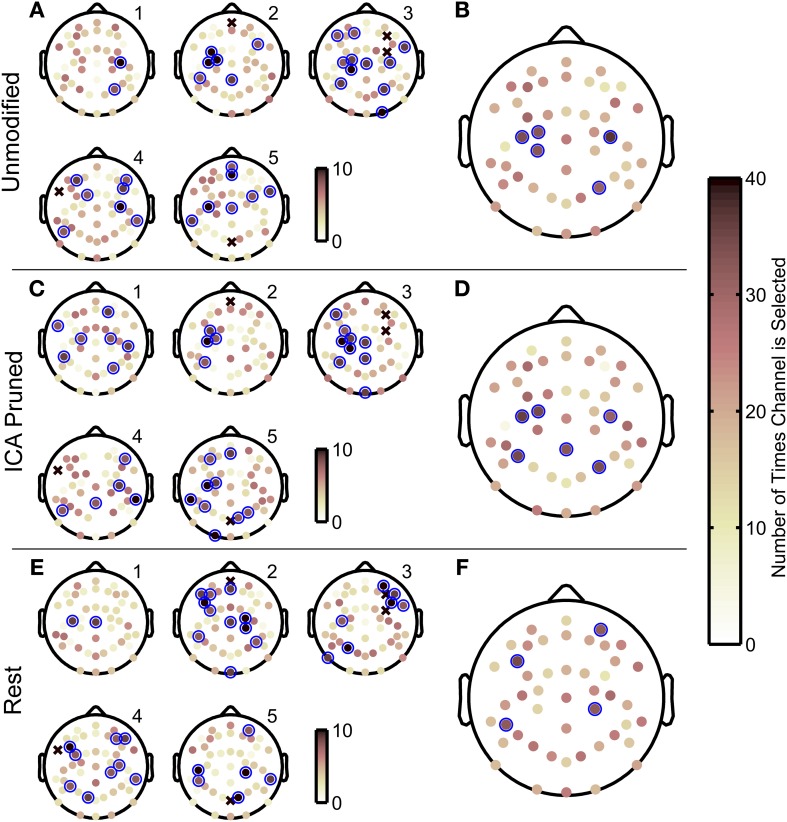
**Scalp maps of the optimal EEG sensor configurations obtained with the genetic algorithm**. Spatial histograms of how often each sensor was selected in optimal sensor sets across the 10 outer cross validation folds for each subject. Histograms pertain to decoding that used unmodified EEG data **(A,B)**, ICA-based artifact removed EEG **(C,D)**, and EEG from resting periods **(E,F)**. The sensor histograms range from 0 to 10 for **(A,C,E)** and range from 0 to 50 for **(B,D,F)**. An x-mark indicates sensors that were manually removed due to poor signal quality. Subject numbers are indicated with labels in the upper right of each scalp map. The sensor histograms in **(B,D,F)** show how often sensors were included in the optimal sensor configuration across all five subjects, where darker circles indicate a sensor that was selected more often. Blue circles mark sensors that were chosen frequently where for individual histograms **(A,C,E)** a sensor is chosen 8 or more times out of 10 genetic algorithm iterations, and for histograms pooled for all subjects **(B,D,F)** sensors were chosen 31 or more times. Assuming a binomial distribution, selecting the same sensor 8 or more times out of 10 trials, or 31 or more times out of 50 trials has a 6% probability occurring by chance.

The GA used in this study converged iteratively to a sensor set that maximized the decoding accuracy for each subject. Figure 7 depicts spatial histograms, plotted on sensor space, the number of times each sensor was included in the optimal sensor sets in all the folds in the *outer* 10-fold cross validation. Panels A,C,E are histograms of chosen sensors within individual subjects. Marked in blue circles are sensors that were picked 8 or more times out of 10 GA optimizations. Panels B,D,F are histograms of chosen sensors grouped across all subjects. Marked in blue circles are sensors that were picked 31 or more times out of 50 GA optimizations. (Assuming binomial distributions, the probability of randomly choosing the same sensor 8 to more times of 10 trials or 31 or more times out of 50 trials were less than 6%).

Interestingly, the scalp electrode distributions with the highest decoding accuracies, corresponding to subjects 2, 3 and 5, included frequently chosen sensors in the contralateral central-medial scalp areas (Figure 7A), whereas the scalp distributions for subjects 1 and 4, who had the lowest decoding accuracies, did not. When pooled together across all subjects, the most frequently chosen sensors were found as a cluster (*N* = 3 sensors) on the central contralateral area, and as two separate sensors on the ipsilateral side (Figure 7B). The spatial histograms were similar for the ICA-pruned EEG data (see Figure 7C). This analysis also showed that the best decoded subjects had a cluster of frequently chosen elctrodes in the central contralateral scalp area. Across all subjects, the most frequently chosen sensors had a sparse distribution near the central regions of the scalp (Figure 7D) When rest EEG was used to decode finger movements, which yielded poor decoding accuracies, the histograms yielded more sparse distributions of frequently chosen sensors. Across all subjects (see Figure 7F), the frequently chosen sensors did not show the cluster of contralateral central electrodes seen in the two prior decoding conditions.

### Spatial distribution of alpha ERD and beta ERS

**Figure 8:**
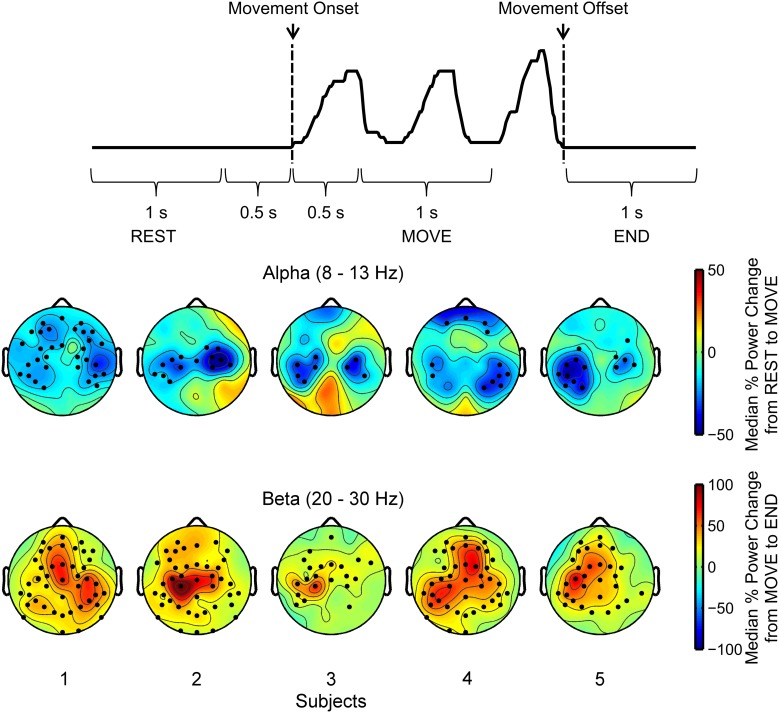
**Scalp maps of alpha ERD and beta ERS across all channels**. The top panel shows the kinematic trace with the three time periods of interest used to calculate alpha ERD and beta ERS that include the rest, movement, and end periods. The five scalp maps in the middle show topographic plots of the median change in alpha band (8–13 Hz) power from rest to movement periods. The five scalp maps in the bottom show topographic plots of the median change in beta band (20–30 Hz) power from movement to end periods. Dots overlaid on the scalp maps indicate channels where alpha ERD and beta ERS calculated across all trials were significantly different (*p* < 0.05) from a distribution with a median of zero based on the sign test.

Figure 8 shows the spatial distribution of the alpha band ERD and beta band ERS. We found that with exception of subject 1, alpha ERDs showed a bilateral distribution near the central-parietal areas of the scalp. Subject 4 also showed alpha suppression during movement near the frontal areas. In regard to beta ERS, subjects 2, 3 and 5 had similar ERS patterns focused on the contralateral central scalp areas, whereas subjects 1 and 4 had distinctive ERS patterns that included frontal areas as well as ipsilateral central-posterior scalp areas. These subjects had the lowest decoding accuracies.

### Spatial and temporal characteristics of delta-band EEG features correlated to kinematics

**Figure 9:**
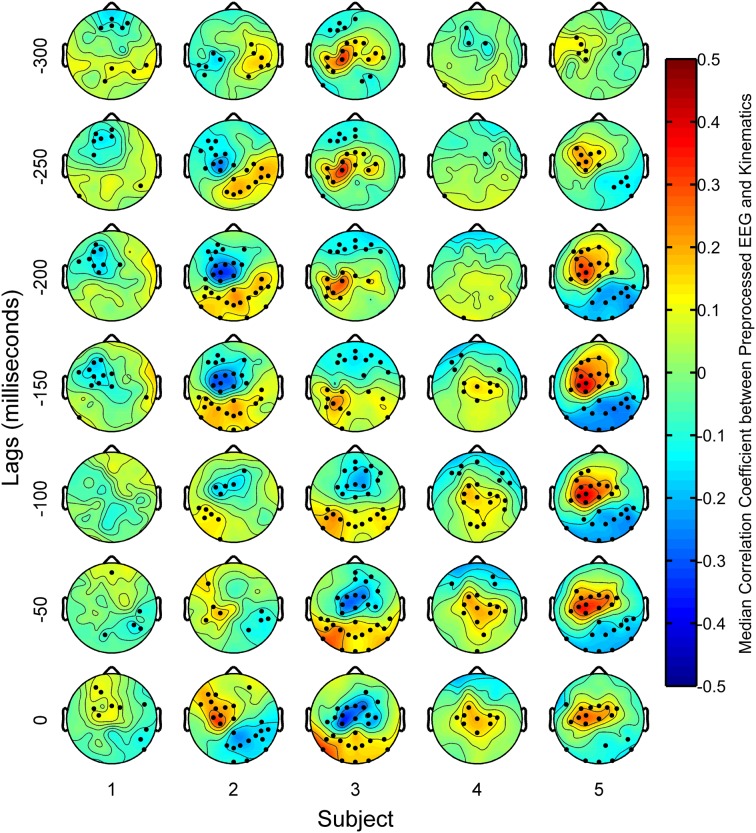
**Scalp maps of median correlation coefficients between the preprocessed EEG features and the preprocessed finger kinematics**. Dots overlaid on the scalp maps indicate channels where the distribution of correlation coefficients across all trials were significantly different (*p* < 0.05) from a distribution whose median was zero based on the sign test.

Figure 9 shows the spatial distribution of sensor areas that were correlated to the finger kinematics. Overall, delta band EEG from subjects 2, 3, and 5 had considerably stronger correlations to finger kinematics than subjects 1 and 4. Subject 2 showed negative correlations with kinematics at contralateral central scalp areas for most lags. For subject 3, whose delta band EEG resulted in the best finger kinematic reconstructions, the scalp maps show that delta band EEG from central-anterior scalp areas at lags from 0 to −100 ms were negatively correlated to finger movements. At lags from −150 to −300 ms, delta band EEG from the central area contralateral to finger movements was positively correlated to the finger movements. Similar patterns were seen for subject 5, except that these positive correlations at the central-medial areas were seen at all lags. Subjects 1 and 4, showed weak correlations at all lags. For subjects 2, 3, and 5, who had moderate to high decoding accuracies, topographies with positive correlations in central areas and negative correlations in the posterior areas (and vice versa) were found at lags with high correlations.

**Figure 10:**
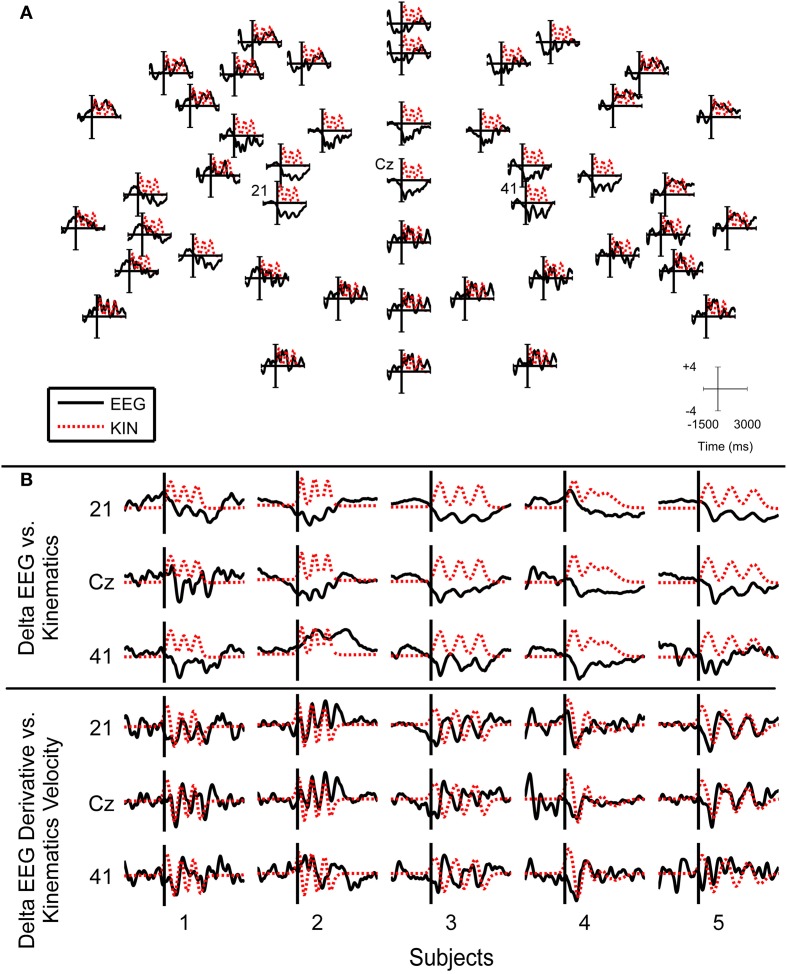
**Grand average delta band (0.1–3 Hz) EEG and finger traces. (A)** The plots show the grand average delta band EEG traces arranged on corresponding sensor locations as indicated in Figure 2B for subject 3. Top sensors correspond to frontal electrodes. Time and amplitude ranges are indicated in axis scale. **(B)** Insets of grand average delta band EEG traces. The first three rows show plots of the grand average delta band EEG for channels 21, Cz, and 41 for each subject. The bottom three rows of plots show plots of the grand average derivative delta band EEG for the respective channels across all subjects. Grand average EEG signals are plotted as solid black lines while grand average kinematic signals are plotted as dotted red lines. All grand averages were standardized to make the kinematics and delta EEG features on the same scale.

Figure 10 shows the grand averages of the delta band EEG and its derivative that was used for reconstructing finger movements in this study. In central areas, the grand average delta band EEG displayed a characteristic increase in negativity. During the movement, the negativity was sustained. At the end of movement, the negativity was reduced back to that of the rest period. During the movement period, the grand average EEG traces resemble that of the grand average kinematics, where about three prominent negative deflections were present. The grand average derivative delta band EEG yielded traces which were more correlated to the grand average kinematics velocities. Such traces were only observed for the subjects 1, 2, 3, and 5 as these subjects performed the finger tapping task with adequate consistency.

## Discussion

### Scalp EEG contains information about finger kinematics that is decodable

The capability of a linear decoder to reconstruct finger movements varied widely across subjects. While decoding accuracies were poor for subjects 1 and 4, they reached moderate values for subjects 2, 3, and 5. It is worth noting that subjects 1 and 4's neural features were different from that of the other subjects. In observing correlations between delta band EEG and kinematics on an individual sensor and lag basis, we observed that delta band EEG from subjects 1 and 4 had considerably less sensors which were significantly correlated, and also had weaker correlations than the other subjects. Subjects 1 and 4 also had considerably wider distributions of alpha ERDs and beta ERSs across the scalp. We cannot offer an explanation of why such neural features from subjects 1 and 4 were different from the other subjects, but they may be linked to the poor decoding accuracies. Previous neuroimaging studies that have studied finger movements also note anomalies in extracting neural features from few subjects (Gerloff et al., 1998b; Ball et al., 1999). It is suspected these anomalies may arise from different mental strategies in performing the task (Ball et al., 1999).

We also found that the standard deviation of the finger tapping speeds was also correlated with the median decoding accuracies across all subjects. Subject 4 had considerably inconsistent kinematics in trial durations and speeds which also resulted in poor grand averages of the kinematics. Our previous work with decoding 3-dimensional reaching kinematics from delta band EEG signals also found a similar finding (Bradberry et al., 2010). While the statistics of finger extension, the resting position, and ROM did not appear to be correlated with accuracies, it should be noted that subject 3 had the smallest ROM and the flattest hand position during rest while subject 2 had the most rounded hand resting position and the smallest finger extensions.

Considering all subjects, the accuracies obtained from this study are low compared to invasive approaches that record brain activity under the dura or intra-cortically using electrode arrays (Kubánek et al., 2009; Acharya et al., 2010; Vargas-Irwin et al., 2010; Zhuang et al., 2010; Bansal et al., 2011; Liang and Bougrain, 2012). Interestingly, Kubánek et al.'s (2009) study reported changes in the LMP over the hand area of motor cortex were associated with flexion of individual fingers. They found that reconstructing finger movements with only LMPs yielded an average accuracy of *r* = 0.40, which is similar to the accuracies found in this study. While it is plausible that the inclusion of other frequency bands may increase decoding accuracies, we limit our analysis to the delta band since alpha and beta band EEG activity have already been extensively studied in neural decoding with EEG with limited success (Blankertz et al., 2010; Hazrati and Erfanian, 2010).

While prior studies are included as a comparison for decoding finger movements, the behavioral tasks from most of these studies were different as they involved grasping and reaching actions. Only Aggarwal et al.'s (2009), Liang and Bougrain's (2012), and Kubánek et al.'s (2009) studies had a behavioral task that called for the movement of individual fingers. Another study from our lab indicated higher decoding accuracies for decoding finger movements during reaching and grasping motions from scalp EEG (Agashe and Contreras-Vidal, 2011). This may indicate a greater difficulty in inferring finger movements from neural activity when the behavioral task calls for individuated finger movements. If separate and more localized areas of the motor cortex are activated in the movement of each individual finger as suggested by fMRI studies (Wildgruber et al., 1997; Beisteiner et al., 2001; Indovina and Sanes, 2001), then it can be speculated that the correlated delta rhythms may be more difficult to extract on the large spatial scale as they arise from smaller and more localized areas of the motor cortex during individual finger movements.

### Considering the possibility of spurious decoding

We argue that EEG artifacts such as eye blinks, eye movements, and muscular activity would not have substantially contributed to the performance of reconstructing finger movements in our study. The behavioral task called for subjects to sit comfortably with the arm resting on the table. Given that the muscles involved in finger extension were located in the forearm, we would argue that they would not have likely influenced the EEG recordings. Also, the decoding was repeated with EEG data that had ocular artifacts removed with ICA, which yielded very similar decoding performances. We also observed that using only the peripheral sensors that included electrodes very close to the eyes and facial muscles yielded poorer decoding accuracies for subjects 2, 3, and 5. Alpha ERDs and beta ERSs were also observed, which indicates a reduction in power in the alpha band during movement and an increase in power in the beta band after movement was finished. If muscular activity had consistently influenced the EEG recordings during the finger movements, an opposite observation would have occurred as electromyography activity tends to contain power in such frequencies. We do note however that the decoding with peripheral sensors in subjects 3 and 5 were higher than decoding with unmodified or ICA-pruned EEG data from subjects 1 and 4. Across all subjects, decoding with peripheral EEG yielded higher accuracies than decoding with rest data. It suggests that correlated information related to finger movements may be embedded in the peripheral sensors to a small extent.

Antelis et al. (2013) argued that the use of linear regression and *r*-values may not be a valid means of measuring the capability of reconstructing limb kinematics from low frequency EEG signals; empirically showing that high *r*-values can be attained from randomly generated data. However, we show that use of rest EEG data to decode finger movements yielded *r*-values centered at *r* = 0 across all subjects, which contradicts that claim. Moreover, an online response to the Antelis et al. study shows that the methods and assumptions on that manuscript may be flawed. The reader is referred to the commentary posted with the publisher for further details.

### Possible mechanisms of improved decoding accuracy through the genetic algorithm

It is known that neighboring EEG sensors have a tendency to be highly correlated with each other. Such correlations occur between neighboring EEG sensors because of the volume conduction of electrical currents arising from the cortex through the tissues of the head. Volume conduction makes it difficult to ascertain which sensors should be added to the decoding analysis. Previous neural decoding studies have employed a neuron dropping analysis, where neurons or sensors that are mostly correlated with movements are added first (Hamed et al., 2007; Bradberry et al., 2009; Acharya et al., 2010). This approach does not readily apply well to EEG due to correlation between neighboring EEG sensors caused by the volume conduction. While some EEG sensors may extract neural activity that is correlated with finger movements, it is difficult to ascertain how much correlated information the sensor may record and how much the sensor is influenced by other uncorrelated neural activity from neighboring cortical tissue. Thus, the use of the GA presents a favorable option in exploring which combination of EEG sensors should be included in the decoding analysis. While the presence of artifacts may not substantially affect the performance of the neural decoding, it may affect the optimal parameters involved in constructing models between EEG and kinematics.

Upon inspection of the optimal sensor sets found with the GA, they appear to have a wide and sparse distribution across the entire scalp, with common and unique sensor selections across subjects. We offer two possible explanations why such a sensor configuration may maximize decoding accuracies. First, the GA may remove EEG sensors that contain little or no neural activity that were correlated with the finger movements. Such sensors may also have a low signal-to-noise ratio. Second, the GA may ‘prune’ the input signals for the decoder by selecting EEG sensors that may have uncorrelated neural activity and removing such components from the other sensors that contain correlated neural activity. From these hypothesized mechanisms, it is expected that EEG sensors in areas of the scalp that contain the most relevant information needed for reconstructing finger movements are most likely to be chosen consistently in the optimal sensor configurations across all subjects. EEG sensors that are chosen to remove uncorrelated or corrupted (i.e., noisy) neural activity from the decoding analysis are expected to have a more random distribution across subjects. Such mechanisms may explain why subjects 2, 3, and 5 had more pronounced clusters in the contralateral central area when ICA-pruned EEG was used in the neural decoding as opposed to unmodified EEG data. Such results may also indicate that artifacts can influence the construction of the model that predicts finger movements from EEG.

### On the use of delta band activity to decode finger movements

Our findings suggest that the contralateral central areas were important for decoding finger movements from delta band EEG activity. We found that EEG sensors that were highly correlated to the finger movements were found in fairly localized areas in central sites of the scalp as shown in Figure 9. Histograms of optimal sensor selections in Figure 7 also suggest that sensors in the contralateral central areas were important for reconstructing finger movements. These findings are consistent with previous finger movement decoding literature, where it has been argued that the motor cortex contains relevant information in decoding finger movements with low frequency neural activity (Kubánek et al., 2009; Acharya et al., 2010; Vargas-Irwin et al., 2010; Zhuang et al., 2010; Bansal et al., 2011). They are also consistent with studies that have found current source activations close to the contralateral central sulcus during finger movements (Gerloff et al., 1998a; Ball et al., 1999; Pollok et al., 2004).

However, we also found that broad posterior areas of the scalp were also correlated as shown in Figure 9. Such areas were usually correlated to finger movements in an opposite manner compared to the contralateral central areas, yielding either central-positive and posterior-negative, or central-negative and posterior-positive topographies. Gerloff et al. (1997) have found similar topographies in EEG voltages during repetitive finger movements, which could be caused by a tangential dipole located in the central sulcus. It may also suggest that sensors needed to decode movements may not necessarily be directly above relevant cortical tissues, which could explain the sparse distribution of sensors in the optimal sensor sets found in the GA.

We also observed that the finger movements yielded a bilateral distribution in alpha ERDs and contralateral beta ERSs as shown in Figure 8, particularly for those subjects that had the highest decoding accuracies. Huang et al. (2004) have demonstrated that dipole sources in ipsilateral regions are from premotor areas and activate earlier than contralateral M1 areas, suggesting they may be involved with inhibitory or planning processes.

In areas of the scalp that were highly correlated to finger movements, the grand averages of the delta band EEG were similar to those of the kinematics as shown in Figure 10. In these grand averages, a characteristic negativity persisted throughout movement, with positive deflections that coincided with the movements. Grand averages of the derivative delta band EEG were also similar to the finger movement velocities. The observation of slow cortical potentials being correlated to movements is relatively new and poorly understood. In recent ECoG studies, these low frequency neural rhythms that are correlated with movements were referred as local motor potentials (Schalk et al., 2007; Kubánek et al., 2009; Acharya et al., 2010). Such local motor potentials were found to have a broad distribution across the cortical mantle (Kubánek et al., 2009), which may be due to volume conduction or the engagement of large ensembles of neural networks during the production of finger movements. It is speculated that these local motor potentials may arise from increased firing rates of neural populations in the motor cortex close to the ECoG electrodes (Schalk et al., 2007; Acharya et al., 2010).

We note there are also non-linearities in neural signatures associated with movements. RPs are often characterized as a slow rise in negativity before movement onset (Cui and MacKinnon, 2009; Bortoletto et al., 2011). Since kinematics do not change during this period, it presents itself as an exemplar that makes it difficult to create a linear relationship between EEG and kinematics. In the grand averages of delta band EEG, we also observed negativity that persisted throughout the movement and diminished when the movement ended. This may explain why our preliminary attempts to reconstruct the position of finger movements from delta band EEG yielded poor decoding accuracies and why using the derivative of delta band EEG to predict finger velocities yielded better results. The negative offset makes it difficult to establish a linear relationship between the EEG and the finger movements. Taking the derivative of the delta band EEG and the finger movements effectively high pass filtered the signals, which caused the sustained negative offset to be suppressed.

## Conclusion

The main finding of this study is that finger kinematics can be inferred, to some extent, from the delta-band filtered fluctuations of the amplitude of EEG signals across the scalp using linear decoders with memory. Cross-validation procedures, including attempts to predict finger kinematics from resting EEG data, and the use of ICA to remove artifactual components from the EEG data, support the main findings. Ancillary analyses on a trial-by-trial basis indicate that delta band EEG is highly correlated to finger movements. Contralateral central areas of the scalp were found to contain high correlations, which is consistent with previous literature relating neural activity to finger movements. The grand averages of the delta band EEG also resembled the finger movements recorded in this study. Decoding accuracies obtained in this study varied across subjects. Interestingly, subjects that showed strong correlations between delta band activity and movement kinematics over contralateral central scalp areas had the highest decoding accuracies whereas subjects with the lowest correlations had the poorest decoding accuracies. Moreover, subjects with a focused bilateral alpha ERDs over central scalp areas and strong beta ERS over contralateral central scalp areas had the highest decoding accuracies as well. Analysis of spatial distributions of EEG sensors selected by the GA also pointed to a cluster of electrodes over contralateral central scalp areas recruited to maximize decoding accuracy. Overall, the methods introduced here may provide a window to study the neural representation of finger movements at the macroscale with scalp EEG.

### Conflict of interest statement

The authors declare that the research was conducted in the absence of any commercial or financial relationships that could be construed as a potential conflict of interest.
